# Regulatory mechanisms and functional analysis of cellular senescence-associated genes GAPDH, CCND1, and HBEGF in the immune microenvironment of meningioma

**DOI:** 10.1186/s12885-026-15593-3

**Published:** 2026-03-17

**Authors:** Jianhuang Huang, Qixiu Wang, Yao Chen, Caihou Lin, Risheng Liang

**Affiliations:** 1 Department of Neurosurgery, Affiliated Hospital of Putian University, Putian, Fujian China; 2 The Affiliated Hospital of Liaoning University of Traditional Chinese Medicine, Shenyang, China; 3 Department of Neurosurgery, Fujian Medical University Union Hospital, Fuzhou, Fujian China

**Keywords:** Meningioma, Cellular senescence, Tumor microenvironment, Single-cell RNA sequencing, Machine learning, Immunotherapy

## Abstract

**Background:**

Meningioma is a common tumor of the central nervous system, yet its pathogenesis remains incompletely understood. Cellular senescence and associated genes play crucial roles in the development of various tumors; however, their functional roles and interactions with the immune microenvironment in meningioma remain poorly characterized.

**Objective:**

This study aims to systematically elucidate the roles of cellular senescence-related genes in meningioma pathogenesis and the tumor immune microenvironment by integrating transcriptomic and single-cell RNA sequencing data, supplemented with in vitro functional validation.

**Methods:**

Publicly available meningioma transcriptomic datasets were analyzed to identify differentially expressed cellular senescence-associated genes (CSA-DEGs). Weighted gene co-expression network analysis (WGCNA) was employed to detect key gene modules. Subsequently, machine learning algorithms, including LASSO regression and random forest, were applied to precisely select CSA-signature genes. Immune infiltration analysis combined with single-cell RNA sequencing data was conducted to characterize macrophage subpopulations and the expression patterns of key genes within the meningioma immune microenvironment. Finally, in vitro gene knockdown experiments were performed to validate the functional roles of GAPDH, CCND1, and HBEGF in meningioma and M2 macrophage polarization.

**Results:**

A total of 43 CSA-DEGs were identified, significantly enriched in pathways related to cellular senescence, the p53 signaling pathway, and inflammatory responses. WGCNA revealed 28 key immune regulatory genes. Immune infiltration analysis showed significant enrichment of M2 macrophages and regulatory T cells in meningioma tissues. Single-cell analysis demonstrated heterogeneity in M2 macrophage populations and differential expression of CSA-signature genes. In vitro knockdown of GAPDH and CCND1 markedly suppressed pro-inflammatory factor expression, whereas HBEGF knockdown reduced anti-inflammatory cytokine secretion by M2 macrophages. These three genes appear to synergistically modulate the immune microenvironment in meningioma.

**Conclusion:**

Cellular senescence-associated genes GAPDH, CCND1, and HBEGF show associations with M2 macrophage polarization and inflammatory cytokine secretion in meningioma. These observations suggest their potential involvement in shaping an immunosuppressive tumor microenvironment and warrant further investigation as possible therapeutic targets.

**Supplementary Information:**

The online version contains supplementary material available at 10.1186/s12885-026-15593-3.

## Introduction

Meningiomas are the most common primary brain tumors in adults [1], presenting significant challenges in clinical management. Despite complete surgical resection, WHO Grade I tumors exhibit a 5-year recurrence rate of 7–23%, while high-grade meningiomas (WHO Grade II/III) have a median progression-free survival of only 2.45–8.43 years even with adjuvant radiotherapy [2]. This high recurrence tendency is closely linked to the inability of current treatments (surgery/radiotherapy) to completely eliminate invasive tumor cells, highlighting an urgent need to investigate the molecular mechanisms of meningiomas to develop novel therapeutic strategies.

In recent years, cellular senescence within the tumor microenvironment has garnered widespread attention. Traditionally regarded as a tumor-suppressive mechanism, emerging studies suggest that senescent cells may promote angiogenesis and immune evasion through the senescence-associated secretory phenotype (SASP) [3, 4]. Previous research has shown that meningioma incidence increases exponentially with age [5], suggesting that the senescent microenvironment may provide unique ecological support for its initiation and progression [6]. Recent findings indicate that p16^INK4A expression is significantly higher in WHO Grade II/III meningiomas compared to Grade I [6], though its specific role and regulatory network remain unclear.

The research offers valuable perspectives on senescence-immune interactions and identifies candidate genes for further validation as potential biomarkers or therapeutic targets. This work aimed to explore novel perspectives that could inform future diagnostic and therapeutic strategies for meningioma. By investigating potential links between cellular senescence and the tumor immune microenvironment, we sought to lay a groundwork for identifying new therapeutic targets, with the ultimate goal of contributing to improved patient outcomes.

## Materials and methods

### Bulk RNA sequencing data analysis

**Fig. 1:**
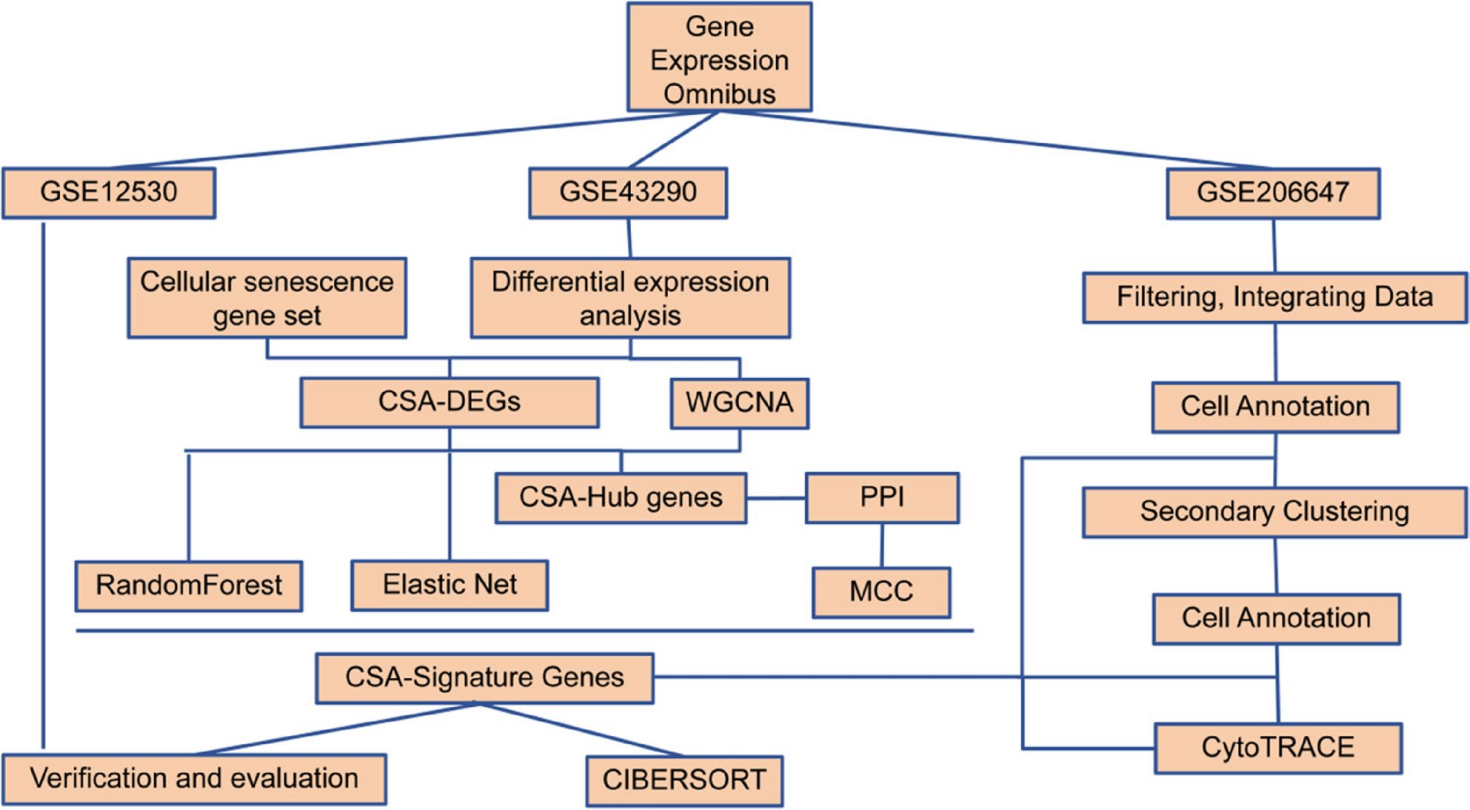
Technical workflow of this study

The technical workflow of this study is illustrated in Fig. 1. Meningioma transcriptomic data were retrieved from the GEO database: ①Keywords: “meningioma” AND (“transcriptome” OR “gene expression”); ② Filters: human samples, including normal controls, with raw data available. Ultimately, GSE43290 (47 tumors/4 normals, GPL96([HG-U133A] Affymetrix Human Genome U133A Array)) was selected as the discovery set, and GSE12530 (24 tumors/2 normals, GPL2895(GE Healthcare/Amersham Biosciences CodeLink Human Whole Genome Bioarray)) as the validation set. Detailed clinical characteristics of samples in each dataset are provided in Supplementary Table S1. This study, based on public database data, involved no human experimentation and complies with Chinese research ethics standards.

### Data preprocessing and quality control

Data standardization was performed using R 4.3.1: ① Chip quality was assessed with the “arrayQualityMetrics” package; ② The “limma” package [7] (v3.58.1) was used for quantile normalization, with batch effect removal verified via principle component analysis (PCA); ③ Gene expression values were log2-transformed, retaining genes detected in ≥ 50% of samples.

### Differential expression analysis (DEGs)

A linear model was constructed using the “limma” package [7] (v3.58.1). DEGs selection criteria were |log2FC| > 1 and false discovery rate (FDR) < 0.05.

### Identification of Cellular Senescence-Associated Differentially Expressed Genes (CSA-DEGs)

A comprehensive set of 503 experimentally validated senescence-related genes was retrieved from the CSGene database (version 2.1). CSA-DEGs were identified by intersecting this senescence gene signature with the previously defined meningioma DEGs using Venn diagram analysis (“VennDiagram” R package, version 1.7.3) [8]. Overlapping genes were considered senescence-associated DEGs for subsequent analyses.

### Weighted Gene Co-expression Network Analysis (WGCNA)

The WGCNA package (v1.72) [9] was used to construct a co-expression network on the GSE43290 dataset. The top 10,000 genes with the highest median absolute deviation (MAD) were selected for analysis. An unsigned network was constructed using the one-step automatic network construction function blockwiseModules with the following parameters: corType = ‘pearson’, soft threshold power β = 4 (selected via pickSoftThreshold function to achieve scale-free topology fit index R² > 0.8 and mean connectivity < 200), minModuleSize = 200, and mergeCutHeight = 0.3. Tumor status was defined as a binary clinical trait (meningioma = 1, normal = 0). Module-trait relationships were assessed by Pearson correlation between module eigengenes (MEs) and clinical traits. The most relevant module was identified based on strong correlation with tumor status (|r| > 0.85 and *P* < 1e-5). This stringent correlation threshold was applied to focus on modules with highly significant and biologically meaningful associations with meningioma while accounting for multiple testing in the context of network analysis.

### Protein-Protein Interaction (PPI) network construction

CSA-DEGs and the most relevant WGCNA module genes were intersected to obtain senescence-related hub genes (CSA-Hub genes). The STRING database V12 (http://string-db.org) was used to construct a PPI network, with a “minimum required interaction score” of 0.7 to ensure reliability. Interaction scores for CSA-Hub genes were imported into Cytoscape V3.9.1 [10] for visualization, and the “cytoHubba” plugin [11] ranked genes using the Maximal Clique Centrality (MCC) algorithm.

### Gene enrichment analysis

The “clusterProfiler” package V4.10 [12] was used for ID conversion and enrichment analysis of differential genes, including Gene Ontology Biological Process (GO_BP), Kyoto Encyclopedia of Genes and Genomes (KEGG) pathway enrichment [13], and Gene Set Enrichment Analysis [14]. Entries with FDR < 0.05 were considered significantly enriched.

### Elastic Net (EN) and Random Forest (RF)

EN and RF models were constructed to identify key genes and perform classification prediction. The dataset was randomly split into training (70%) and testing (30%) sets using the ‘caret’ package [15], with oversampling via the ‘themis’ package [16] to address class imbalance. Both models were trained within the ‘caret’ framework, employing 10-fold cross-validation (repeated 3 times) for hyperparameter tuning. For EN, parameters (alpha and lambda) were optimized using ‘glmnet’ [17]; for RF, mtry and other parameters were tuned with tuneLength = 5. Final model performance was evaluated on the hold-out test set, with ROC curves and AUC values generated using the ‘pROC’ package [18].

### Identification of CSA-signature genes

The top 20 genes from Random Forest, Elastic Net, and MCC algorithms were intersected to identify CSA-signature genes, visualized using Venn diagrams [8].

### Immune infiltration analysis

Although the LM22 reference matrix of CIBERSORT was originally derived from peripheral blood leukocytes [19, 20], it has been extensively validated for accurately estimating immune infiltration proportions in various solid tumor tissues, including gliomas and breast cancer. Given the absence of a dedicated tissue-specific immune reference profile for meningiomas, the continued use of LM22 remains appropriate.The “CIBERSORT” algorithm assessed immune cell abundance in meningiomas and controls, calculating Pearson correlation coefficients and P-values (FDR < 0.05 deemed significant) between CSA-signature genes, immune cell abundance, and immune checkpoints.

### Single-cell data preprocessing and cell type identification

Single-cell data were sourced from GEO dataset GSE206647 (18 meningioma samples, 2 normal meninges, platform GPL24676). Analysis was performed using the “Seurat” package [21] (v5.1.0), with quality control criteria: 200 < nFeature_RNA < 6000, mitochondrial content < 10% (excluding apoptotic cells). Batch effects were removed using the “Harmony” package [22] V1.2.0. Count matrices were normalized and scaled using Seurat’s “NormalizeData” and “ScaleData” functions. Clustering resolution was set to 0.6, with UMAP for dimensionality reduction and cell annotation based on known markers [23]. Macrophage single-cell data were extracted for secondary dimensionality reduction, clustering, and annotation.The marker genes for microglia are CX3CR1, P2RY12, and TMEM119. The marker genes for M1 macrophages are IL1B, CD86, and TNF. The marker genes for M2 macrophages are MRC1, CD163, and MSR1 [23].

### CytoTRACE analysis

CytoTRACE (version 0.3.3) [24] was applied to the normalized single-cell expression matrix of macrophage subsets using default parameters (ncores = 8, subsamples = 1000). Analysis was restricted to M2-like macrophage clusters extracted from the full dataset. Stemness/differentiation scores were calculated for each cell, and transcriptional activity was inferred from gene count correlations. Differences in CytoTRACE scores between clusters were assessed using Wilcoxon rank-sum test with Bonferroni correction for multiple comparisons.The “ggplot2” package [25] explored pseudotime expression changes of CSA-signature genes.

### Cell lines and culture

The human meningioma cell line Ben-Men-1 (Shanghai Cell Bank) was cultured in Dulbecco’s Modified Eagle Medium (DMEM, Gibco) supplemented with 10% fetal bovine serum (FBS, Gibco, heat-inactivated at 56 °C for 30 min) and 1% penicillin-streptomycin (Baitai Bio). Cells were maintained at 37 °C in a humidified incubator with 5% CO₂. Experiments were conducted using cells in the logarithmic growth phase. Passaging was performed at a 1:3 split ratio every 2–3 days with medium replacement. The human monocytic cell line THP-1 (Adigene) was cultured in RPMI-1640 medium (Gibco) under the same conditions.

### THP-1 macrophage differentiation and polarization

THP-1 cells were seeded in 6-well plates at a density of 1 × 10⁶ cells/well and treated with phorbol 12-myristate 13-acetate (PMA, 100 ng/mL; Sigma-Aldrich) for 24 h to induce differentiation into M0 macrophages. After induction, the medium was replaced with PMA-free medium and cells were rested for 24 h. To generate M1 macrophages, M0 cells were treated with lipopolysaccharide (LPS, 100 ng/mL; Sigma-Aldrich) combined with interferon-gamma (IFN-γ, 20 ng/mL; PeproTech) for 24 h. For M2 polarization, M0 macrophages were treated with interleukin-4 (IL-4, 20 ng/mL; PeproTech) and interleukin-13 (IL-13, 20 ng/mL; PeproTech) for 24 h.

### SiRNA design and transfection

siRNAs targeting GAPDH, CCND1, and HBEGF were purchased from GenePharma (Shanghai, China). The sense strand sequences were as follows:

si-GAPDH: 5’-GAAGGTGAAGGTCGGAGTCA-3’, si-CCND1: 5’-GAGCUAUGUGUGUGGAAUU-3’, si-HBEGF: 5’-GCGGUUGUGUGAUGAAUAA-3’A non-targeting siRNA was used as negative control (si-NC). Ben-Men-1 cells and polarized macrophages were transfected with 50 nM siRNA using Lipofectamine 3000 (Invitrogen, Thermo Fisher Scientific) according to the manufacturer’s instructions. Cells were harvested 48 h post-transfection for RNA extraction and subsequent functional assays. Transfection efficiency was validated by RT-qPCR to confirm target gene knockdown.

### RNA extraction and quantitative PCR

Total RNA was extracted using TRIzol reagent (Invitrogen) following the manufacturer’s protocol. RNA purity and concentration were measured by NanoDrop 2000 (Thermo Scientific), ensuring A260/A280 ratios between 1.8 and 2.0. Complementary DNA (cDNA) was synthesized with PrimeScript RT reagent kit (Takara) using 1 µg of total RNA in a 20 µL reaction volume. Quantitative PCR was performed on a QuantStudio 5 system (Applied Biosystems) using SYBR Green PCR Master Mix (Takara) in 20 µL reactions. Primers spanning exon-exon junctions were used (primer sequences provided in Table S2). The cycling conditions were: initial denaturation at 95 °C for 30 s, followed by 40 cycles of 95 °C for 5 s and 60 °C for 34 s. GAPDH expression was normalized to β-actin, which was validated as a stable reference gene under experimental conditions using geNorm analysis. Relative gene expression was calculated using the 2^-ΔΔCt method, normalized to β-actin. The stability of β-actin as a reference gene was confirmed by consistent Ct values across experimental conditions (variation < 1 cycle).

### Cytokine ELISA

Cell culture supernatants were collected 48 h after transfection or treatment and centrifuged at 1000 × g for 10 min. The concentrations of IL-10 and TGF-β in the supernatants were measured using human ELISA kits (R&D Systems) according to the manufacturer’s protocols. Samples and standards were assayed in triplicate. Absorbance was read at 450 nm using a BioTek microplate reader, and cytokine concentrations were calculated from standard curves fitted by a four-parameter logistic model.

### Statistical analysis

All statistical analyses were performed using R software (version 4.3.1). Experimental procedures were independently repeated at least three times. Data are presented as mean ± standard deviation (SD) for normally distributed variables or median with interquartile range (IQR) for non-normal data, as appropriate.For two-group comparisons: Student’s t-test (parametric) or Wilcoxon rank-sum test/Mann-Whitney U test (non-parametric).For multiple group comparisons: one-way ANOVA with Tukey’s post-hoc test (parametric) or Kruskal-Wallis test with Dunn’s post-hoc test (non-parametric).Correlation analyses: Pearson correlation coefficient for normally distributed data; Spearman rank correlation otherwise. Multiple testing correction: Benjamini-Hochberg FDR adjustment was applied where appropriate. *P*-values < 0.05 were considered statistically significant (*P < 0.05, **P < 0.01, ***P < 0.001, ****P < 0.0001).

## Results

### Transcriptomic features of meningiomas and senescence-related gene screening

**Fig. 2:**
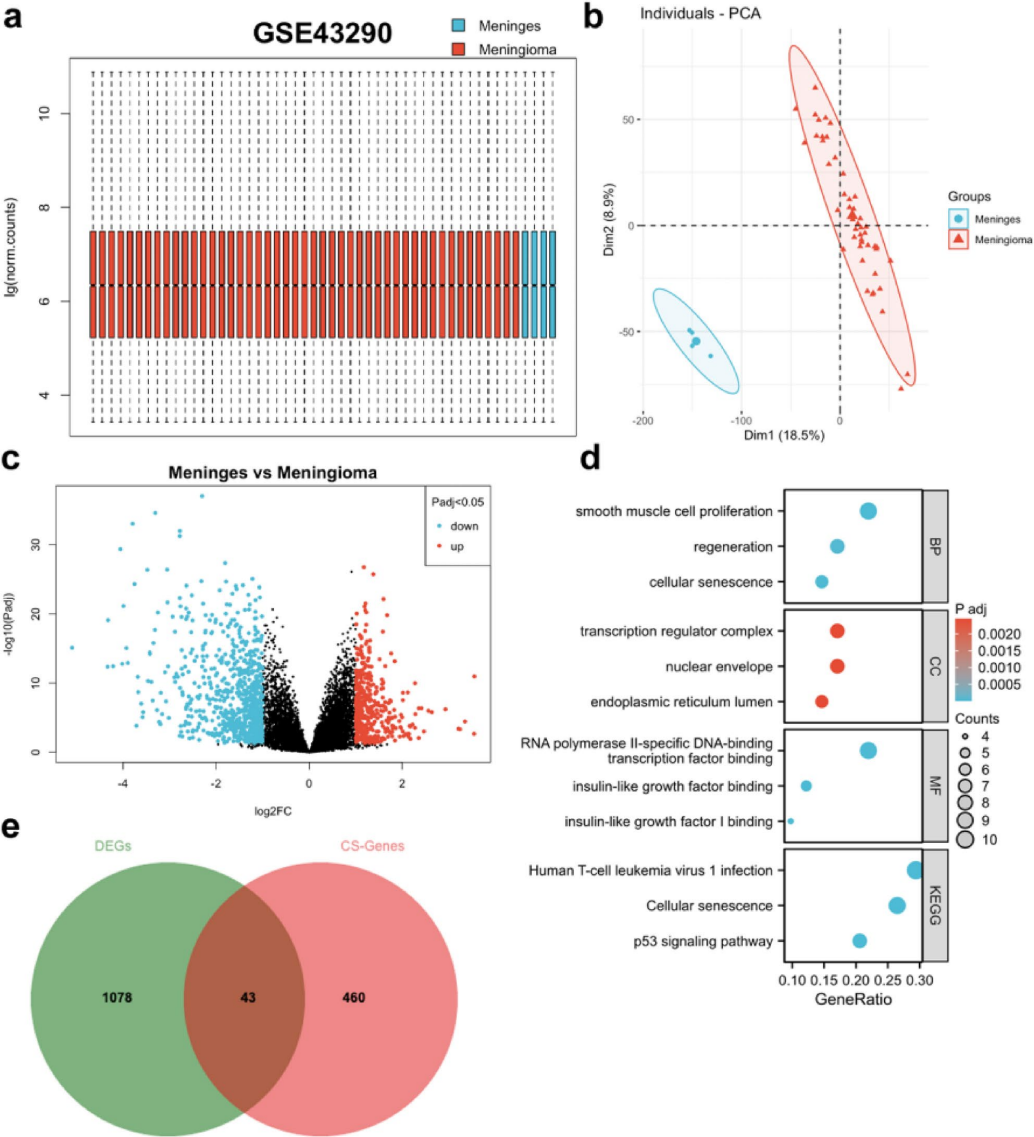
Identification and Functional Enrichment Analysis of Cellular Senescence-Related Differentially Expressed Genes. **a**-**b** Quality control status of the GSE43290 data. **c** Results of differential expression analysis in GSE43290. **d** Bubble chart displaying the functional enrichment analysis results of cellular senescence-related differentially expressed genes. **e** Venn diagram showing the identification of cellular senescence-related differentially expressed genes

The GSE43290 dataset demonstrated good data quality (Fig. 2a), with significant gene expression differences between groups (Fig. 2b). Differential expression analysis identified 1121 DEGs (Fig. 2c). Functional enrichment analysis revealed pathways including p53 (FDR = 8.31857E-07) and cellular senescence (FDR = 8.31857E-07) (Fig. 2d). Intersection with 503 senescence-related genes identified 43 CSA-DEGs (Fig. 2e).

### WGCNA identifies core modules in co-expression network

**Fig. 3:**
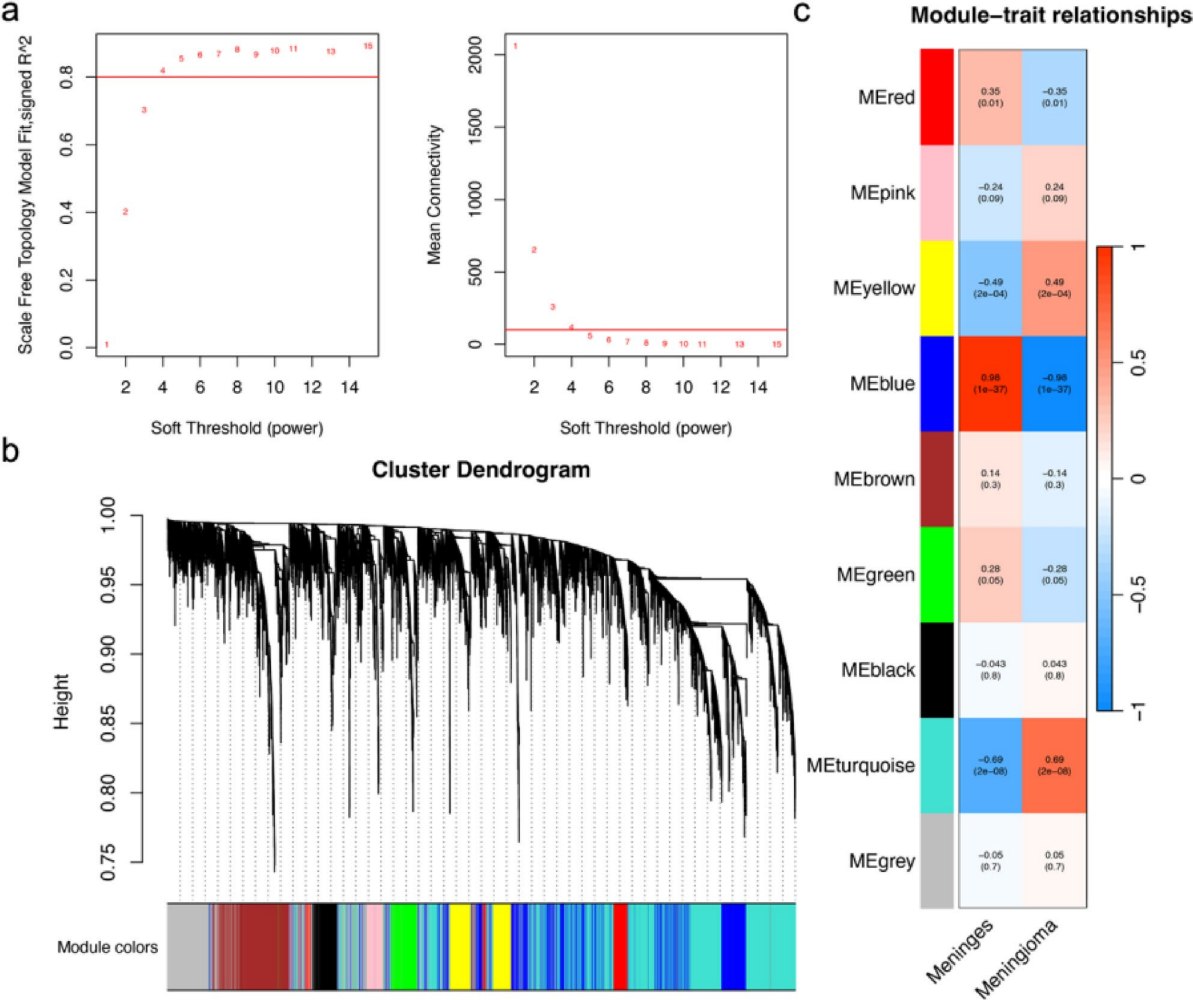
Identification of Key Modules in WGCNA. **a** Based on the results of the pickSoftThreshold function, the optimal soft-threshold power is set to 4. **b** A co-expression network was constructed using the one-step method, identifying 9 modules. **c** The heatmap shows the correlation between gene expression within modules and module eigengenes. WGCNA, Weighted Gene Co-expression Network Analysis

A co-expression network was constructed using standardized GSE43290 data, with good quality. The optimal soft threshold was set to 4, identifying 9 modules via dynamic tree cutting (Fig. 3a-b). The MEblue module (1,713 genes, Table S3) was highly correlated with tumor phenotype (*r* = 0.98, *P* = 1 × 10⁻³⁷, Fig. 3c). Uniform gene connectivity within the module suggested robust network construction.

### PPI network and functional hub gene screening

**Fig. 4:**
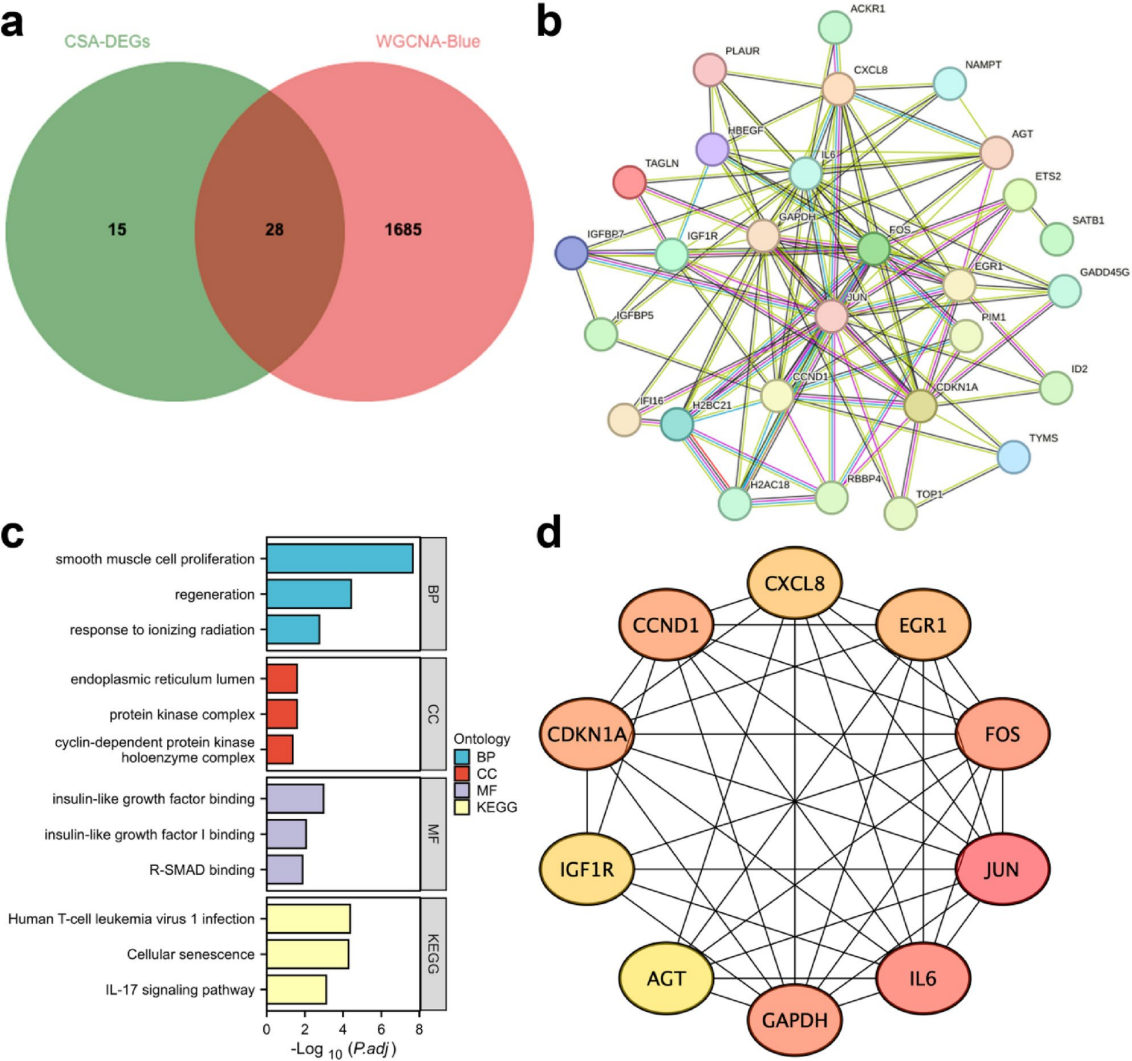
PPI Network Analysis and Gene Functional Enrichment. **a** Venn diagram showing the intersection of CSA-DEGs and the WGCNA blue module genes. **b** PPI network constructed from the intersected genes. **c** Functional enrichment analysis results of the intersected genes. **D** Bar chart displaying the top 10 genes ranked by score using the MCC algorithm, with darker colors indicating higher scores. PPI, Protein-Protein Interaction.PPI, Protein-Protein Interaction.CSA-DEGs, Cellular Senescence-Associated Differentially Expressed Genes. WGCNA, Weighted Gene Co-expression Network Analysis.MCC, Maximal Clique Centrality

Intersection of CSA-DEGs with WGCNA blue module genes yielded 28 CSA-Hub genes (Fig. 4a, Table S4). A PPI network constructed via STRING contained 28 nodes and 111 edges (Fig. 4b). Reliability validation showed a median STRING interaction score of 0.82, exceeding random expectation (*P* = 1 × 10⁻⁵). Enrichment analysis of CSA-Hub genes included cellular senescence (FDR = 5.20803E-05), p53 signaling (FDR = 0.003), and IL-17 signaling (FDR = 0.0003) (Fig. 4c). MCC algorithm identified top 10 hub genes, including CCND1 and GAPDH (Fig. 4D).

### Machine learning model construction and biomarker validation

**Fig. 5:**
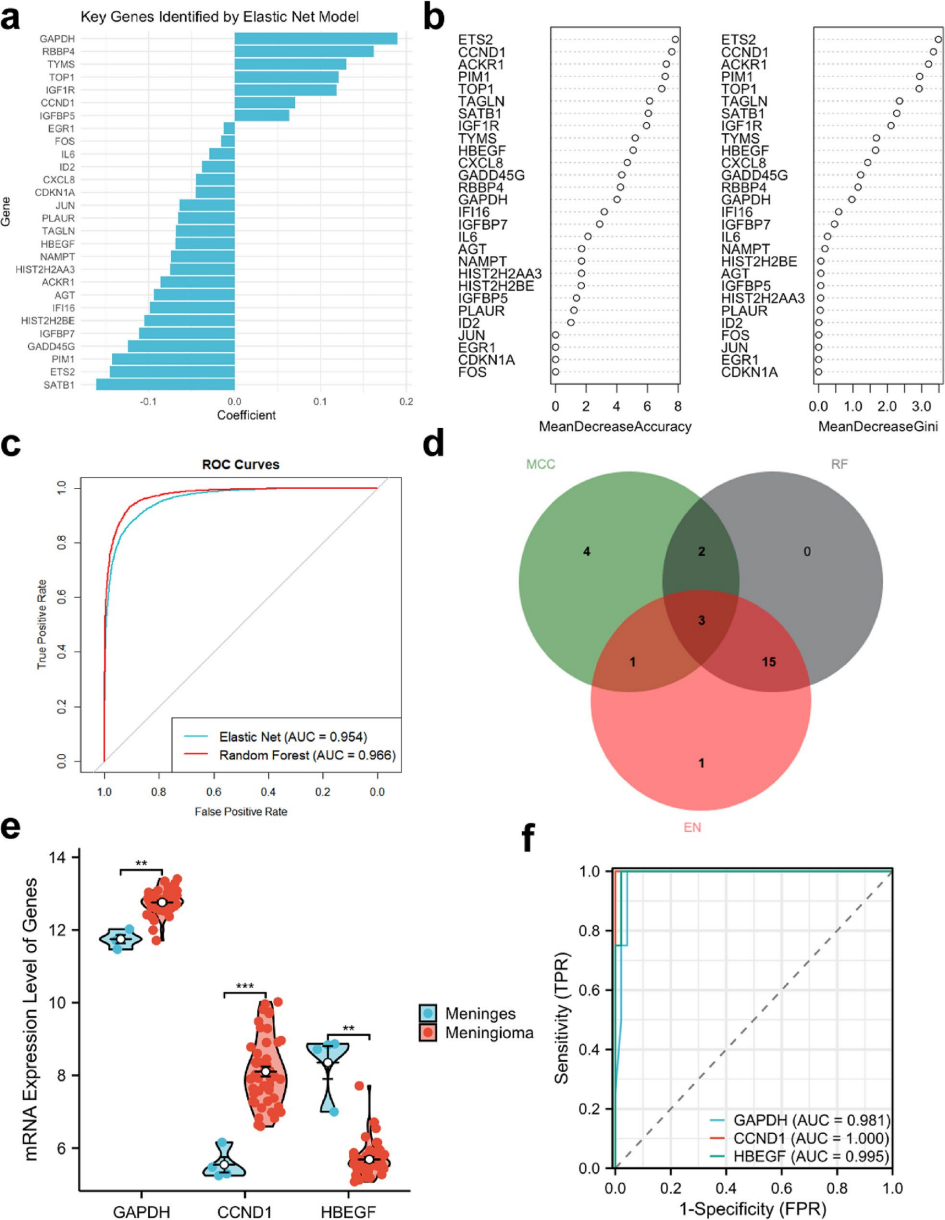
Identification and Validation of CSA-signature Genes. **a** Bar chart showing the top 20 CSA-Hub genes ranked by importance in the elastic net model. **b** Bubble chart displaying the top 20 CSA-Hub genes ranked by importance in the random forest model. **c** ROC curves showing the performance of the elastic net model and random forest model. **d** Venn diagram showing the intersection of top 20 CSA-Hub genes ranked by MCC algorithm, elastic net model, and random forest model, identifying three CSA-signature genes. **e** Comparison of expression levels of three CSA-signature genes in different samples in the GSE43290 dataset. **f** ROC curves displaying the diagnostic performance of the three CSA-signature genes in the GSE43290 dataset. CSA, Cellular Senescence-Associated. MCC, Maximal Clique Centrality

Elastic Net and Random Forest models identified the top 20 CSA-DEGs by importance (Fig. 5a-b). AUC values for distinguishing normal meninges from meningiomas were 0.954 and 0.966, respectively (Fig. 5c-d). Integration of MCC, Elastic Net, and Random Forest models identified 3 CSA-signature genes: GAPDH, CCND1, and HBEGF (Fig. 5e, Table S5). In GSE43290, GAPDH and CCND1 were significantly upregulated in meningiomas (*p* < 0.01 and *p* < 0.001), while HBEGF was downregulated (*p* < 0.01), with excellent diagnostic performance (AUC = 0.981, 1, and 0.995, Fig. 5f). Results were consistent in the external validation set (Figure S1).

### Immune microenvironment remodeling and macrophage polarization

**Fig. 6:**
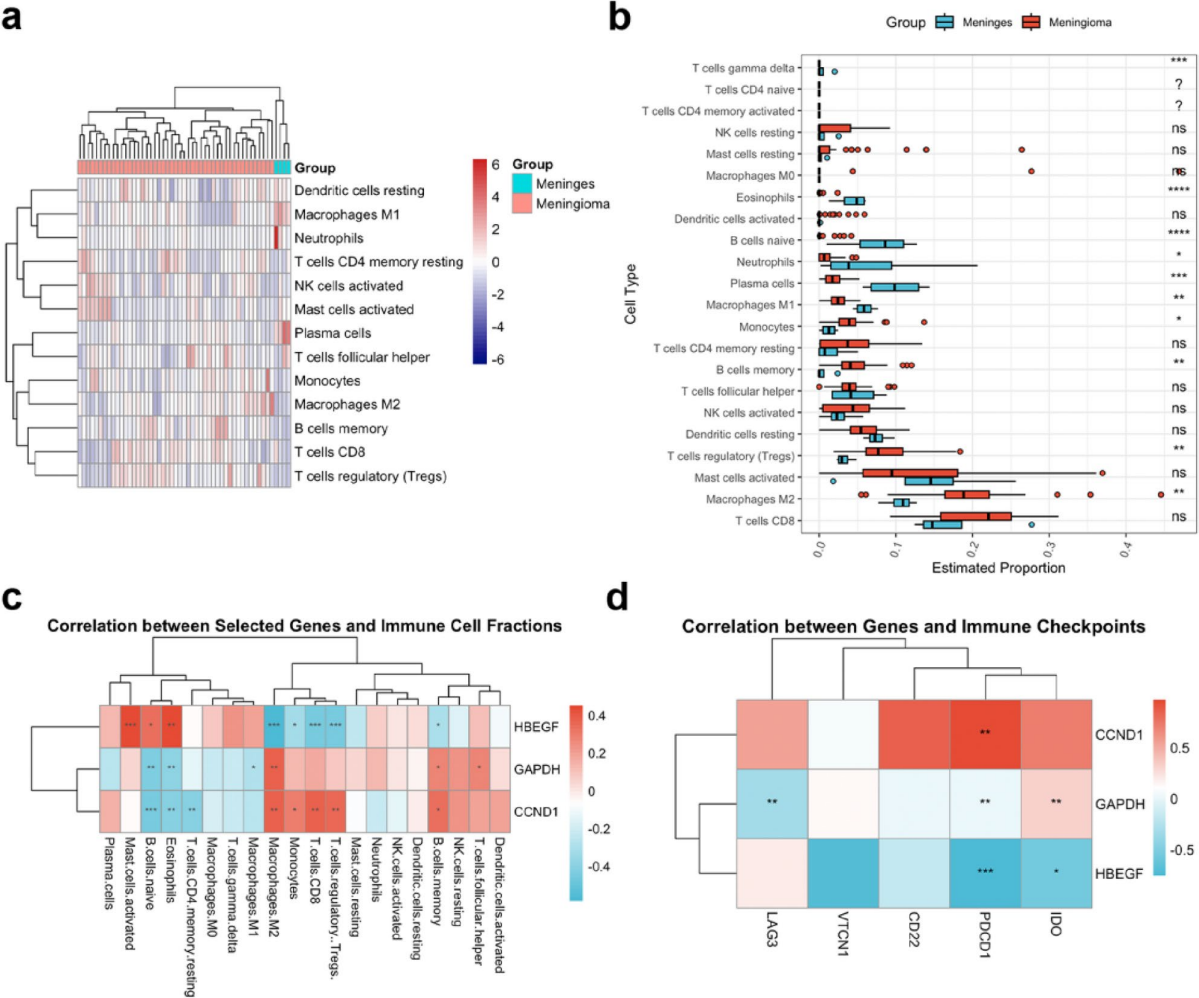
Immune Infiltration Analysis of Meningiomas. **a** Heatmap showing significant differences in immune heterogeneity between the two groups. **b** Bar chart comparing the abundance of immune cells between meningiomas and normal meningeal tissues. **c**-**d** Heatmap displaying the correlation between the three CSA-signature genes and immune cell abundance, as well as immune checkpoints

CIBERSORT analysis revealed significantly higher abundances of M2-like macrophages, Tregs, and memory B cells in meningiomas compared to controls (FDR < 0.05, Fig. 6a-b). Correlation analysis showed M2 macrophage abundance positively correlated with GAPDH (*r* = 0.49, *p* < 0.01) and CCND1 (*r* = 0.48, *p* < 0.01) expression, and negatively with HBEGF (*r* = −0.44, *p* < 0.001,Fig. 6c). Upregulation of GAPDH and CCND1 may promote M2 polarization via metabolic reprogramming or cell cycle regulation, while HBEGF downregulation may weaken its immunostimulatory role. CD8 T cell abundance positively correlated with CCND1 (*r* = 0.47, *p* < 0.01) and negatively with HBEGF (*r* = −0.45, *p* < 0.001), with no significant correlation with GAPDH (Fig. 6c). Additionally, CCND1 (*r* = 0.54, *p* < 0.01) and HBEGF (*r* = −0.58, *p* < 0.001) showed significant correlations with PDCD1 levels (FDR < 0.01, Fig. 6d).

### Single-cell resolution analysis of tumor heterogeneity

**Fig. 7:**
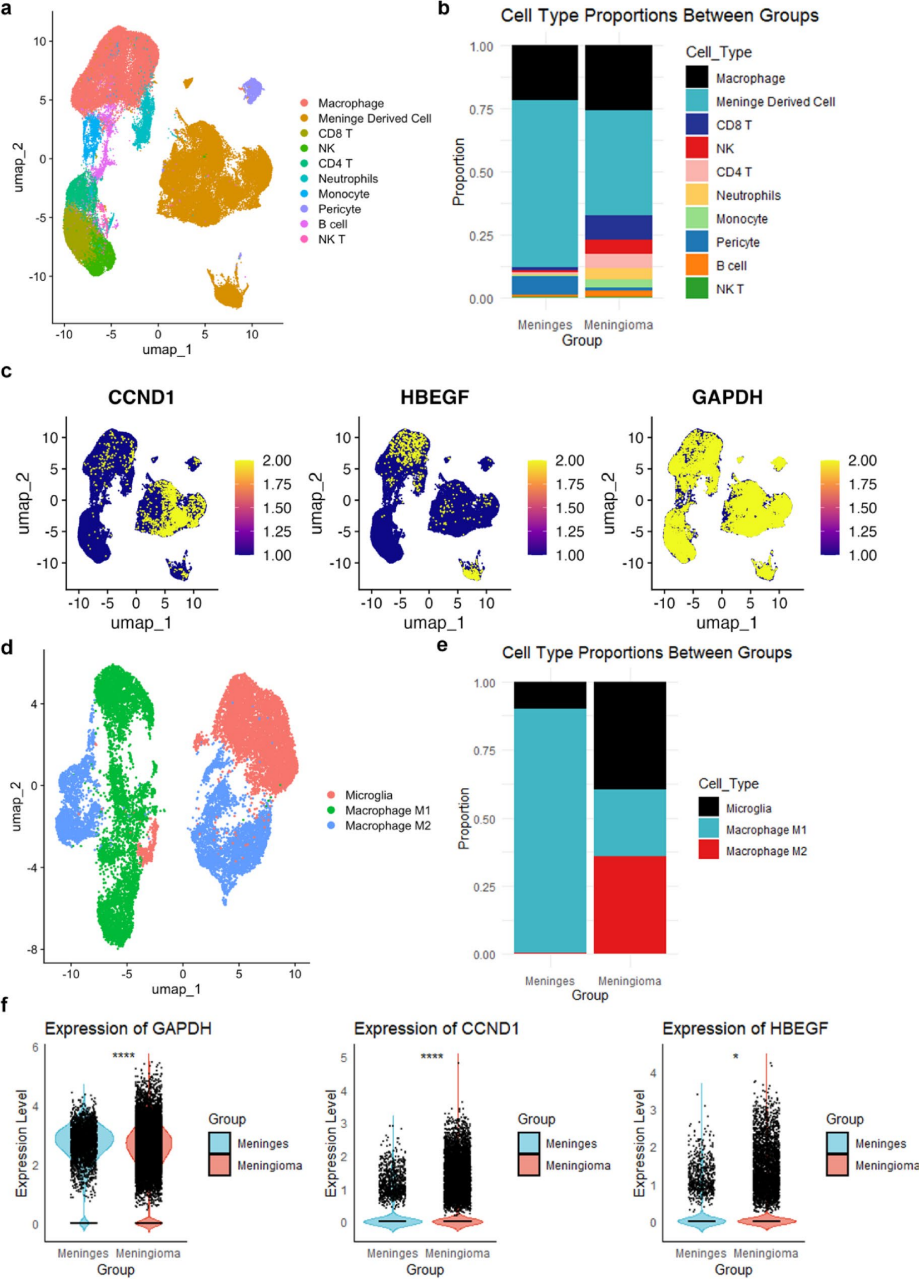
Single-Cell Analysis. **a** Classification of all subclusters into 10 major cell types based on known cell markers. **b** Comparison of cell proportions between meningiomas and normal meningeal tissues. **c** Expression patterns of CSA-signature genes at the single-cell level. **d** Secondary clustering analysis of macrophage single-cell data, with a resolution of 0.5, identifying 14 different subclusters. **e** Bar chart comparing the proportion of M2-like macrophages between meningiomas and normal meningeal tissues. **f** Comparison of CSA-signature gene expression in macrophages

Single-cell RNA sequencing identified 24 distinct subclusters (Figure S2A-B), annotated into 10 major cell types based on known markers (Fig. 7a). Group comparisons showed higher proportions of macrophages (26.01% vs. 22.13%) and CD8 T cells (9.90% vs. 1.18%) in meningiomas, with reduced meningeal-derived cells (66.03% vs. 41.39%) (Fig. 7b), suggesting a key role for immune cells in meningioma development. CSA-signature gene expression analysis revealed widespread GAPDH expression across cell types, elevated CCND1 in meningeal-derived cells, and high HBEGF in macrophages, potentially linked to its immune response role (Fig. 7c). Given the potential importance of macrophages, secondary dimensionality reduction and clustering (resolution = 0.5) identified 14 subclusters (Figure S2C), annotated into 3 major types (Fig. 7d). We validated expression using dot plots of cell-type–specific marker genes, and dot plots for the top marker genes of each cell subpopulation are shown in Supplementary Figure S2D. Notably, M2-like macrophages were significantly higher in meningiomas (36.0% vs. 0.03%) (Fig. 7e). Compared to bulk RNA-seq, meningioma macrophages exhibit upregulation of CCND1, HBEGF, and GAPDH (FDR < 0.0001, < 0.05, and < 0.0001, respectively; Fig. 7f), which is consistent with the bulk RNA-seq findings.

### CytoTRACE analysis

**Fig. 8:**
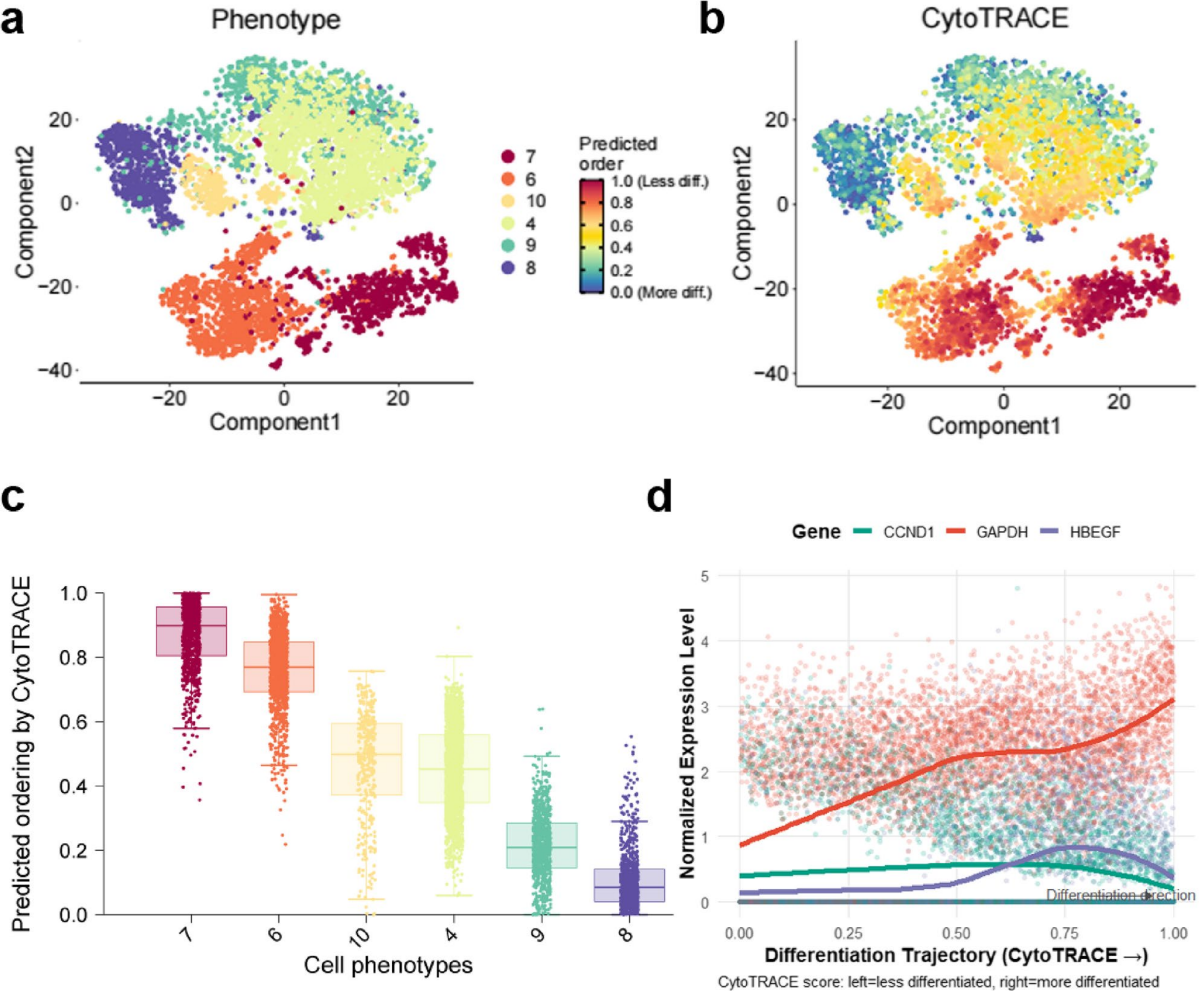
CytoTRACE analysis. **a** t-SNE plot showing M2 macrophage clusters. **b**-**c** Transcriptional activity distribution of M2 macrophages. **d** Expression changes of CSA-signature genes during M2 macrophage differentiation.

CytoTRACE analysis revealed significant heterogeneity in inferred stemness among M2 macrophage subclusters(Cluster 4,6,7,8,9,10). Cluster 7 exhibited the highest stemness score (indicating less differentiated, more plastic state), while cluster 8 showed the lowest score (more terminally differentiated) (Fig. 8a–c). Pseudotime trajectory reconstruction demonstrated progressive differentiation along the inferred path: GAPDH expression was markedly upregulated in the early phase of M2 polarization. HBEGF showed peak expression in mid-pseudotime In contrast, CCND1 maintained relatively stable expression throughout the trajectory(Fig. 8 d).

### Expression of CSA-signature genes and their regulatory effects

**Fig. 9:**
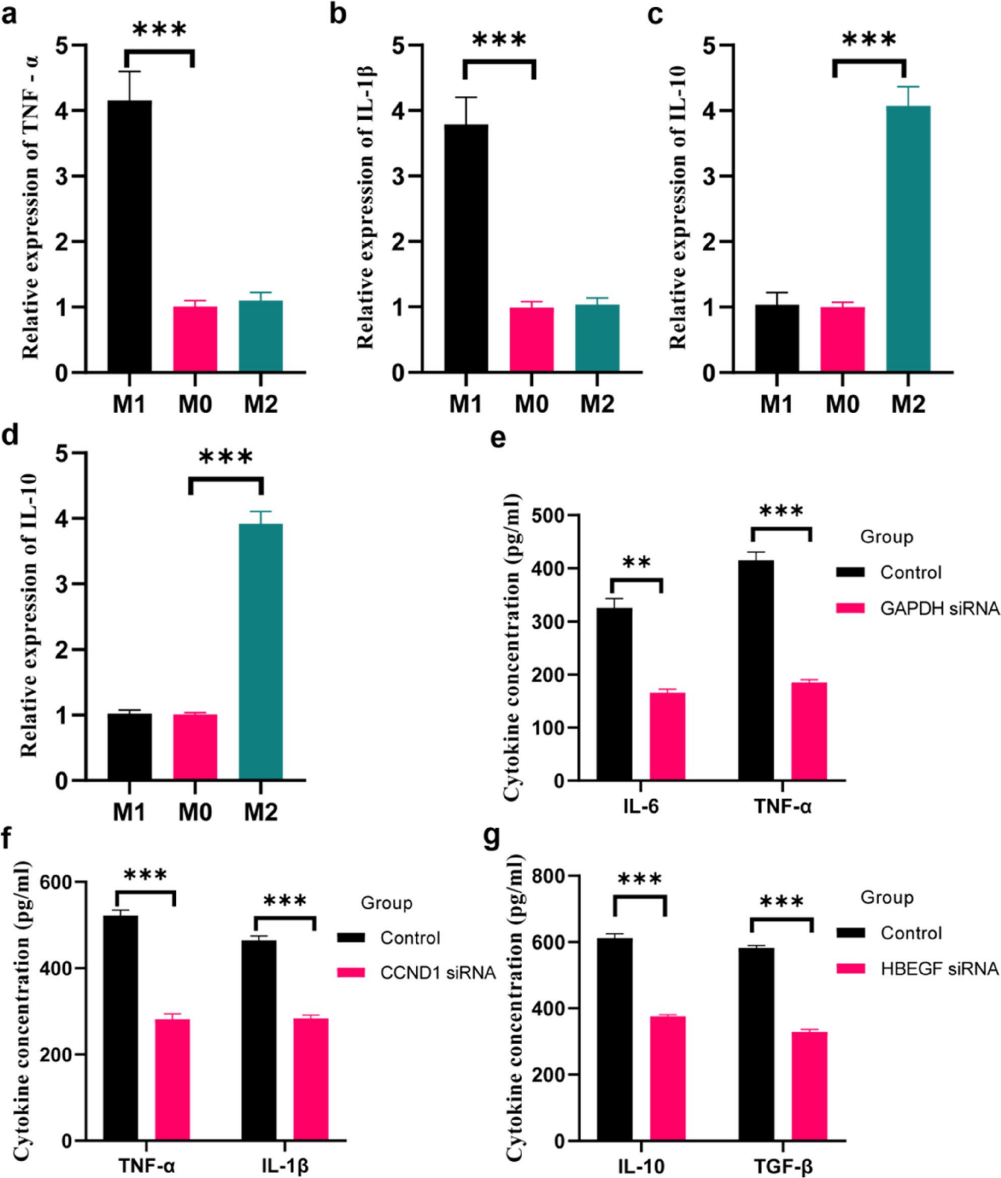
Expression of CSA-signature genes and analysis of their regulatory effects. **a**-**b** RT-qPCR detection of M1 polarization markers in THP-1 monocytes. **c**-**d** RT-qPCR detection of M2 polarization markers in THP-1 monocytes. **e** Significant reduction of IL-6 and TNF-α concentrations in the culture supernatant of Ben-Men-1 cells following GAPDH knockdown. **f** CCND1 knockdown significantly inhibited TNF-α and IL-1β secretion in M1-polarized THP-1 macrophages. **g** HBEGF knockdown significantly decreased IL-10 and TGF-β secretion in M2-polarized THP-1 macrophages.

THP-1 monocytes were induced into M0 macrophages by PMA treatment, followed by polarization into M1 and M2 phenotypes using LPS + IFN-γ and IL-4 + IL-13, respectively. RT-qPCR results showed that pro-inflammatory cytokines TNF-α and IL-1β were significantly upregulated in the M1 group compared to the M0 group (*p* < 0.001, Figs. 9a-b), confirming successful induction of pro-inflammatory M1 macrophage polarization. In the M2 group, anti-inflammatory cytokines IL-10 and TGF-β were markedly increased (*p* < 0.001, Figs. 9c-d), validating the establishment of the M2 polarization model.

SiRNA-mediated knockdown of GAPDH in the meningioma cell line was effective, and ELISA measurement of inflammatory cytokines in the culture supernatant revealed significant reductions in IL-6 and TNF-α concentrations in the GAPDH knockdown group (*p* < 0.001, Fig. 9e). In the M1-polarized THP-1 macrophage model, knockdown of CCND1 led to a significant decrease in the secretion of pro-inflammatory cytokines TNF-α and IL-1β, as measured by ELISA (*p* < 0.001, Fig. 9f). Similarly, in the M2-polarized THP-1 model, knockdown of HBEGF significantly reduced the secretion of anti-inflammatory cytokines IL-10 and TGF-β (*p* < 0.001, Fig. 9g).

## Discussion

In this study, through integration of transcriptomic and single-cell analyses combined with in vitro functional validation, we investigated the roles of cellular senescence-associated genes GAPDH, CCND1, and HBEGF in meningioma tumorigenesis and its immune microenvironment. The observed gene expression patterns and their functional outcomes reveal a complex and context-dependent regulatory landscape, where apparent discrepancies between correlation analyses and direct perturbation assays underscore the multifaceted roles of these genes.The upregulation of CCND1 is consistent with its known role in cell cycle regulation in other cancers; however, its direct impact on proliferation in meningioma was not functionally assessed here. The dynamic expression of HBEGF in M2 macrophages suggests its potential involvement in shaping an immunosuppressive microenvironment, consistent with prior reports on its functions in inflammation and tumors. Compared with previous studies [23, 26], our results further highlight the potential contribution of M2 macrophages to meningioma progression, suggesting new opportunities for immunomodulatory therapies targeting the tumor microenvironment.This interpretation is supported by the observed enrichment of M2-like cells in our analysis and existing evidence linking M2 polarization to tumor advancement [27], although direct validation in larger, prospective cohorts is required.

Differential expression analysis revealed 43 CSA-DEGs enriched in pathways related to cellular senescence, p53 signaling, and inflammatory response, reflecting the dual role of senescence in cancer. While senescence can limit tumor growth, it may also facilitate progression through secretion of pro-inflammatory SASP factors that remodel the tumor microenvironment [28]. It has been suggested that some tumors evade immune surveillance by modifying senescent phenotypes, promoting progression [29]. Similar mechanisms could potentially operate within the meningioma microenvironment, though our study does not provide direct evidence for this.

WGCNA identified 28 CSA-Hub genes enriched in immune regulation and inflammatory responses, implying their potential involvement in meningioma development via modulation of the immune microenvironment. Immune infiltration analysis showed increased immunosuppressive cells, including M2 macrophages, regulatory T cells (Tregs), and memory B cells, with decreased M1 macrophages and activated CD4⁺ T cells in meningiomas.M2 macrophages are known for their pro-tumorigenic effects, suppressing T cell activity and promoting immune evasion through anti-inflammatory cytokines and inhibitory molecules like PD-L1 [30]. Our data indicate functional heterogeneity in tumor-associated macrophages in meningioma, potentially exerting dual roles in tumor progression and suppression [31]. The predominant M2-like subsets may facilitate immune escape by fostering an anti-inflammatory milieu, whereas M1-like subsets could, in certain contexts, exert suppressive effects through mechanisms such as antigen presentation. This plasticity, potentially influenced by SASP factors, highlights a possible avenue for therapeutic intervention via reprogramming of immunosuppressive subsets.

In vitro experiments on the CSA-signature genes demonstrated distinct yet complementary roles of GAPDH, CCND1, and HBEGF in meningioma cells and macrophage polarization. Although traditionally viewed as a housekeeping gene, GAPDH exhibits noncanonical pro-tumorigenic functions in various cancers, often related to metabolic reprogramming and oxidative stress regulation [32]. In this study, GAPDH knockdown in meningioma cells reduced secretion of pro-inflammatory cytokines IL-6 and TNF-α, suggesting a potential role for tumor cell-derived GAPDH in sustaining a local pro-inflammatory state.This appears to contrast with its positive correlation with immunosuppressive M2 macrophages in bulk tissue analysis. However, this discrepancy may reflect GAPDH’s pleiotropic functions: in tumor cells, it could drive a SASP-like inflammatory response, while in the tumor microenvironment, its metabolic or signaling functions might indirectly support the recruitment or polarization of M2 macrophages.Beyond its glycolytic function, GAPDH displays moonlighting roles independent of metabolism [33, 34], including nuclear translocation for gene and cell cycle regulation, extracellular signaling, oxidative stress response, and inflammatory pathway modulation [35, 36]. This multifunctional nature—maintaining core metabolism while possibly enabling context-dependent immune effects—makes GAPDH a candidate of interest. Its association with senescence-related inflammation in tumor-associated macrophages further supports exploring its role in tumor-immune crosstalk. However, future studies using specific inhibitors or conditional models are needed to dissect these functions in meningioma.

CCND1, encoding cyclin D1, regulates the G1/S transition and is linked to enhanced proliferation and poor prognosis in many cancers [37]. Notably, while CCND1 expression correlated positively with M2 macrophage abundance, its knockdown in M1-polarized macrophages inhibited secretion of pro-inflammatory cytokines TNF-α and IL-1β.This implies a potential, non-cell-cycle-related role in inflammatory macrophage responses. HBEGF, a growth factor expressed in inflammatory settings, influences macrophage activation and differentiation [38]. Its upregulation in macrophages implies a possible role in modulating the tumor immune microenvironment. HBEGF knockdown reduced secretion of immunosuppressive cytokines IL-10 and TGF-β in M2 macrophages, underscoring its apparent importance in M2 polarization and immunosuppressive activity. This functional validation suggests that HBEGF acts as a direct positive regulator of M2 immunosuppressive function. The negative correlation in bulk analysis might be explained if HBEGF expression is highly specific to a subset of tumor-associated macrophages rather than being broadly upregulated across all M2 cells in the tumor bulk, or if its expression is subject to feedback regulation within a fully established immunosuppressive niche.

Integration with immune infiltration data showed correlations between these genes and M2 abundance, hinting at their coordinated, though likely complex, effects on the meningioma immune landscape. Furthermore, the observed correlation between CSA-signature genes and PDCD1 expression suggests a possible link to immune checkpoint regulation, which could reflect aging-associated T cell exhaustion. This finding is consistent with observations in other cancers and provides a rationale for investigating combinations of senescence-modulating approaches with immune checkpoint blockade; however, direct mechanistic evidence in meningiomas is lacking and requires further validation.

Single-cell RNA sequencing showed increased M2-like macrophages with functional heterogeneity in meningiomas. The differential expression of CSA-signature genes across macrophage subsets supports the idea that they may play roles in macrophage differentiation and diversity, aligning with the known plasticity of M2 cells in tumors [39]. These observations underscore the value of further investigating interactions between macrophage subsets, senescence-associated genes, and meningioma progression.

This study has several strengths: (1) integration of multi-omics data with experimental validation to examine senescence-associated genes in meningioma; (2) identification and functional assessment of GAPDH, CCND1, and HBEGF as potential core modulators of the immune microenvironment; (3) provision of evidence supporting a role for M2 macrophages in meningioma immune evasion, offering a foundation for future immunotherapy research. However, several limitations warrant consideration. First, the sample size is limited and largely derived from public datasets, with most samples representing WHO grade I meningiomas; higher-grade and recurrent cases are underrepresented. This may bias the observed senescence–immune interactions and limit generalizability. Second, while in vitro functional validation was performed, it relied on a single Ben-Men-1 cell line and lacks in vivo models, patient-derived systems, and clinical sample validation. Proliferation assays and pathway inhibitors were not employed to corroborate the proposed mechanisms. Third, immune infiltration analysis depended on CIBERSORT with the LM22 reference, which may not fully capture features of meningeal-resident macrophages and can be affected by technical factors. Fourth, we did not directly assess cellular senescence markers or SASP secretion; consequently, the impact of SASP on the immune microenvironment remains inferential. Future work should incorporate larger, more diverse cohorts and integrate advanced models and spatial multi-omics to further validate these mechanisms and their clinical relevance.

## Conclusion

This study investigates the roles of senescence-associated genes GAPDH, CCND1, and HBEGF in meningioma tumorigenesis and immune microenvironment regulation, indicating links between cellular senescence and tumor immune evasion. These findings offer insights into meningioma progression and potential for senescence-targeted therapies. The work highlights senescence-immune interactions and identifies candidate genes for further evaluation as biomarkers or therapeutic targets.

## Supplementary Information

Supplementary Material 1.

Supplementary Material 2.

## Data Availability

GSE43290: https://www.ncbi.nlm.nih.gov/geo/query/acc.cgi?acc=GSE43290 GSE12530: https://www.ncbi.nlm.nih.gov/geo/query/acc.cgi?acc=GSE12530 GSE206647: https://www.ncbi.nlm.nih.gov/geo/query/acc.cgi?acc=GSE206647 Cell Senescence Database: http://csgene.bioinfo-minzhao.org

## Abbreviations

CSA-DEGs: Cellular Senescence-Associated Differentially Expressed Genes
WGCNA: Weighted Gene Co-expression Network Analysis
PPI: Protein-Protein Interaction
MCC: Maximal Clique Centrality
GO_BP: Gene Ontology Biological Process
KEGG: Kyoto Encyclopedia of Genes and Genomes
GSEA: Gene Set Enrichment Analysis
EN: Elastic Net
RF: Random Forest
ROC: Receiver Operating Characteristic
AUC: Area Under the Curve
CSA: Cellular Senescence-Associated
FDR: False Discovery Rate
PCA: Principal Component Analysis
MAD: Median Absolute Deviation
scRNA-seq: Single-Cell RNA Sequencing
