# Supplementary material for: Regulatory mechanisms and functional analysis of cellular senescence-associated genes GAPDH, CCND1, and HBEGF in the immune microenvironment of meningioma
Source: BMC Cancer. 2026 Mar 17;26:524. doi: 10.1186/s12885-026-15593-3 (PMC13107606; doi:10.1186/s12885-026-15593-3)
Supplement: Supplementary file 2 — Supplementary Material 2. [file 12885_2026_15593_MOESM2_ESM.docx]

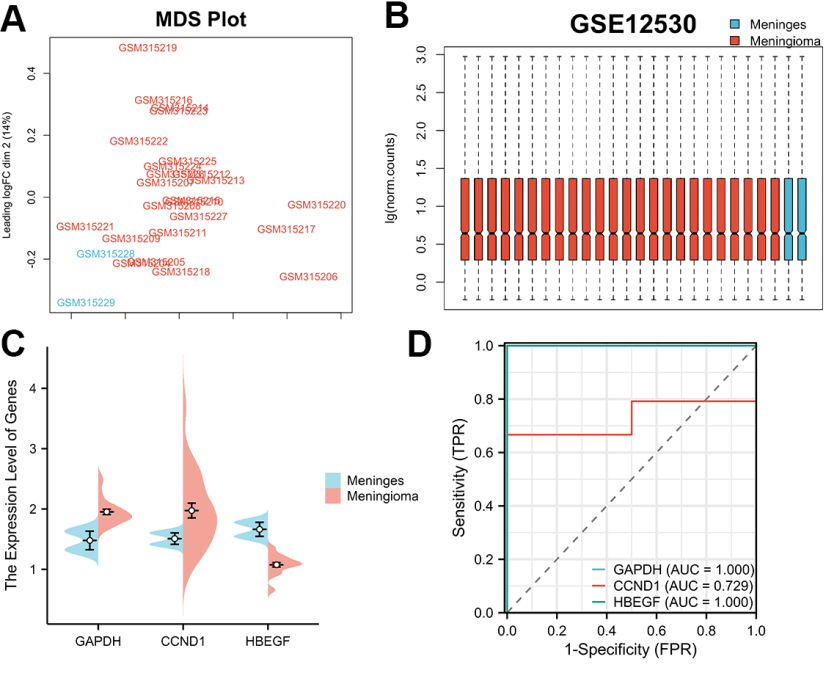


Figure S1. External validation of CSA-signature genes.A. MDS plot showing the distribution of samples.B. Box plot displaying data quality control results.C. Expression differences of CSA-signature genes in meningioma and normal meningeal samples.D. ROC curve demonstrating the diagnostic potential of CSA-signature genes.


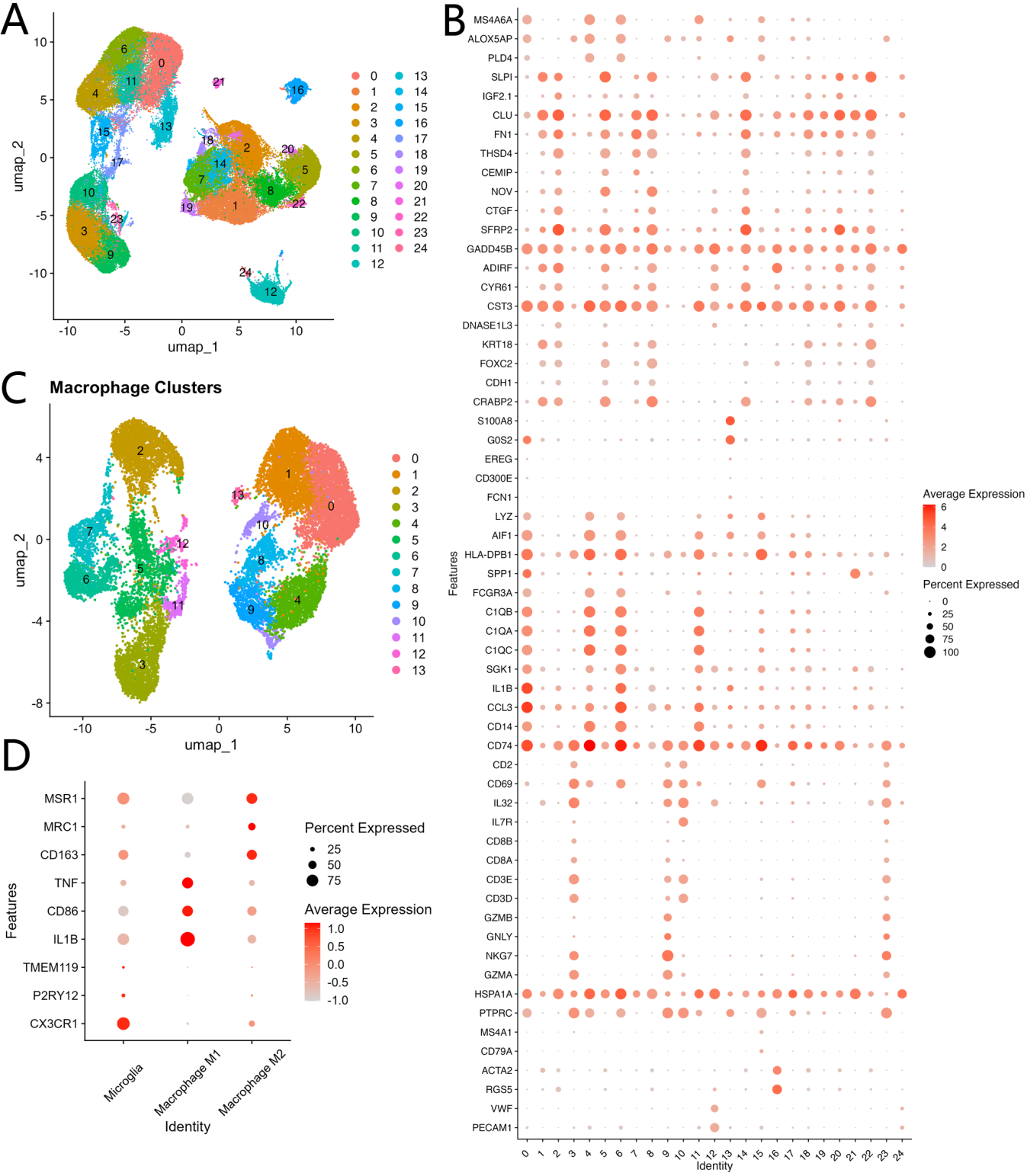


Figure S2. Single-cell clustering and marker gene expression.

A–B. Clustering and marker gene expression in meningioma versus normal meninges (single-cell analysis).

C–D. Clustering and marker gene expression in macrophage subpopulations identified in a secondary analysis.
